# Testing social cognition in multiple sclerosis: Difference between emotion recognition and theory of mind and its influence on quality of life

**DOI:** 10.1002/brb3.1925

**Published:** 2020-11-01

**Authors:** Matthias Grothe, Michael Opolka, Julia Berneiser, Alexander Dressel

**Affiliations:** ^1^ Department of Neurology University Medicine of Greifswald Greifswald Germany; ^2^ Department of Neurology Carl‐Thiem‐Hospital Cottbus Cottbus Germany

**Keywords:** multiple sclerosis, quality of life, social cognition

## Abstract

**Background:**

Deficits in social cognition can occur in multiple sclerosis (MS) patients, and different methods are utilized for its assessment. The aim of this study was to compare two tests of social cognition in a cohort of multiple sclerosis patients with respect to other clinical variables. Additionally, the impact of social cognition on quality of life was investigated.

**Methods:**

In total, 50 patients were included in the study. Two tests of social cognition, emotion recognition and theory of mind, were performed and controlled for disease disability, depression, fatigue, and cognition in a multiple linear regression. Assessment of quality of life was also conducted.

**Results:**

Accuracy on emotion recognition was better compared to theory of mind (86.5 ± 9.5% and 63.6 ± 10.1%, respectively). Cognition was associated with both social cognition tasks, accounting for more variance in the emotion recognition task. Quality of life was not related to social cognition.

**Conclusion:**

Studies on social cognition in MS have to keep in mind the higher degree of cognitive influence of emotion recognition compared to theory of mind.

## Abstract 2

Social cognition in multiple sclerosis can be assessed with emotion recognition and theory of mind. Cognition effects emotion recognition more than theory of mind. Social cognition impairment is not associated with quality of life.

## 1 INTRODUCTION

Neuropsychological impairment in multiple sclerosis (MS) is increasingly gaining interest (Langdon, 2011; Prakash et al., 2008). There is a broad literature about cognitive impairments in MS, especially on information processing, working memory, and executive function (Langdon, 2011; Langdon et al., 2012), but only little attention has been made to more social cognitive functions. Social cognition (SC) with its different aspects of processing, deciding, or responding to social stimuli plays an important role in everyday life (Frith, 2008), and many neurological disorders can be accompanied by SC deficits (Henry et al., 2016). Usually, SC is assessed with either emotion recognition (ER) or theory of mind (TOM) tasks (Henry et al., 2015). ER tasks investigate the ability to *identify* emotional states, whereas TOM tasks require *assigning* emotional states. One of the most often used TOM test to judge social cognitive functioning is the Read the Mind in the Eyes (RMIE) task (Baron‐Cohen et al., 2001). Compared to other SC tasks, RMIE is considered as low cognitive demanding (Henry et al., 2015).

In MS, several disease‐related clinical variables potentially confound SC impairment. While some studies suggested an influence of cognitive impairment on SC (Henry et al., 2009), other studies did not find such an association (Berneiser et al., 2014; Roca et al., 2008). Comparable discrepancies were reported for associations between SC and disease disability (Banati et al., 2010; Henry et al., 2011), fatigue (Berneiser et al., 2014; Henry et al., 2011), and depression (Berneiser et al., 2014; Phillips et al., 2011). These inconsistencies might be due to varying methodological approaches like the choice of psychological assessments (i.e., ER vs. TOM), different sample sizes, and differences in the number of confounders that were taken into account. A summarizing meta‐analysis proved the association between cognitive decline and SC impairment, while depression was not related to (Bora et al., 2016).

There is a broad literature about neuropsychological domains and their influence on quality of life (QOL) (Benito‐Leon et al., 2002; Lysandropoulos et al., 2015), but only limited evidence about the effect of SC impairment (Phillips et al., 2011).

Here, we tested different SC tasks (ER and TOM) in one sample of MS patients. We were interested in clinical factors (i.e., clinical status, cognition, depression, and fatigue) that might influence SC ability. We hypothesized that different SC tasks are differentially associated with those factors, especially with regard to cognition.

Additionally, we focused on QOL, testing the hypothesis that impaired QOL is related to SC disabilities for both, ER and TOM tasks.

## 2 MATERIALS AND METHODS

### 2.1 Participants

Patients with a diagnosis of multiple sclerosis according to the 2017 McDonald criteria (Thompson et al., 2018) consulting the neurological outpatient clinic of the Greifswald university hospital were asked to participate. In total, 50 patients were included consecutively in the study after giving their written informed consent. Exclusion criteria were an acute relapse or steroid therapy within 30 days prior to enrollment, a history of psychiatric disorders other than depression, visual acuity <0.8, motor inability in performing the task, and other central nervous system diseases. The study was approved by the local ethics committee of the medical faculty of the University of Greifswald (BB08/13).

### 2.2 Assessment

Disability status was rated using the Expanded Disability Status Scale (EDSS) (Kurtzke, 1983), depression was assessed using the Beck Depression Inventory (BDI) (Beck & Steer, 1987), and fatigue using the Fatigue Scale for Motor and Cognitive Functions (FSMC) (Penner et al., 2009). Additionally, participants were requested to complete the Multiple Sclerosis International Questionnaire of Quality of Life (MusiQoL), a disease‐specific, international validated questionnaire evaluating nine dimensions (activity of daily living (ADL), psychological well‐being (PWB), symptoms (SPT), relationships with friends (RFr), relationships with family (RFa), relationships with healthcare system (RHCS), sentimental and sexual life (SSL), coping (COP), and rejection (REJ)) and yielding a global index score (Flachenecker et al., 2011; Simeoni et al., 2008). All dimension scores were linearly transformed to a 0–100 scale. The global index score was computed as the mean of the dimension scores.

Cognitive function was assessed using the Brief Repeatable Battery (BRB) (Rao, 1990; Scherer et al., 2004), a well‐known and broadly applied battery for the assessment of cognitive functions in MS. The BRB consists of several subtests assessing verbal memory (selective reminding task [SRT]), visual memory (spatial recall test [SPART]), semantic fluency (word list generation [WLG]), information processing speed, and working memory (paced auditory serial addition test [PASAT], symbol digit modalities test [SDMT]) (Rao, 1990). A total BRB z‐score was calculated based on the raw values from the subtests (Scherer et al., 2004).

SC was investigated with two different tasks: For ER, a facial morphing task was used with a face depicting one out of four expressions (happy, angry, fearful, sad), morphing from neutral to the target expression in a 10‐s morphing sequence (100 pictures with 100‐ms duration each with increasing valence of the expression) (Lischke et al., 2012). Participants had to press a button when they presumably had recognized the expression. After pressing the button, a screen was displayed where the participants had to choose the target expression out of four proposed emotions (happy, angry, fearful, and sad). In total, 48 morphing sequences in random order were shown, 12 for each expression (Lischke et al., 2012). Accuracy as the percentage of correct recognized expressions was recorded. For TOM, we used a computer‐based version of the RMIE (Baron‐Cohen et al., 2001). In this task, participants were asked to infer the mental states from a photograph depicting the eyes of a face choosing one of four proposed alternatives. In total, 36 pictures were presented in a 20‐s time window and the accuracy (as percentage) recorded. Cognitive and SC assessment order was counterbalanced, half the participants starting with the BRB, half with the SC tasks, and SC tasks either beginning with ER or TOM.

### 2.3 Statistical analysis

All data were analyzed with IBM SPSS Statistics 21. Data were expressed as the means/standard deviations (*SD*) or the medians/ranges, depending on the parametric or nonparametric distribution of the variable. For each SC task, multiple linear regression was calculated separately using accuracy as dependent variable (ER or TOM), and disability (EDSS), depression (BDI), fatigue (FSMC), and cognition (BRB) as independent variables without ordering.

For the QOL measurements, Pearson's correlations were calculated between SC results (ER; TOM) and global MusiQoL score as well as subscores. If correlating significantly, their association with the same predictors (EDSS, BDI, FSMC, BRB) was assessed by performing a multiple regression analysis. Statistical significance was defined as *p* < .05.

Basic assumptions of normality were assessed performing Shapiro–Wilk test.

## 3 RESULTS

Sample characteristics with means/median of cognitive ability, fatigue, depression, and disability are shown in Table 1. Patients were mildly disabled with a median EDSS of 2.0., moderately fatigued (*M* = 59.4, *SD* = 19.8) and minimally depressed (*M* = 9.7, *SD* = 8.9). Their cognitive status proved to be on average (*M*=−0.3, *SD* = 1.4).

**TABLE 1:** Demographic and clinical characteristics

Disease course (RRMS/SPMS/PPMS)	44/5/1
Age (years)^a^	39.4 ± 9.7
Sex (male/female)	21/29
Education (years)^a^	13.9 ± 2.2
BRB^a^, ^c^	−0.3 ± 1.4
FSMC^a^	59.4 ± 19.8
BDI^a^	9.7 ± 8.9
EDSS^b^	2.0 (1–7.5)

Abbreviations: BDI, Beck Depression Inventory; BRB, Brief Repeatable Battery; EDSS, Expanded Disability Status Scale; FSMC, Fatigue Score for Motor and Cognitive Functions; PPMS, primary progressive multiple sclerosis; RRMS, relapsing–remitting multiple sclerosis; SPMS, secondary progressive multiple sclerosis.

^a^ Means ± standard deviations.

^b^ Median (range).

^c^ Results are given as *z*‐values of the total BRB‐N.

Mean accuracy was 86.5% (9.5) for ER and 63.6% (10.1) for the TOM task.

Multiple linear regression for ER and TOM reached significance (*F*(4,49) = 11.73; *p *< .001; *F*(4,49) = 4.08; *p *= .007, respectively). For both SC tasks, only cognition predicted SC ability, with a higher beta value for ER (beta = 0.57; *p* < .001) than for TOM (beta = 0.37; *p* = .01). For details, see Table 2.

**TABLE 2:** Multiple linear regression models for SC tasks

Model	*R*	*R* ^2^	*R* ^2^ adjusted	*F*	Variable	Beta	*p*
ER	0.72	0.51	0.47	11.74			<.001
					BDI	0.19	.14
					FSMC	−0.18	.17
					BRB	0.57	<.001
					EDSS	−0.17	.15
TOM	0.52	0.27	0.20	4.08			.007
					BDI	0.15	.33
					FSMC	0.09	.56
					BRB	0.37	.01
					EDSS	−0.20	.15

Abbreviations: BDI, Beck Depression Inventory; BRB, Brief Repeatable Battery; EDSS, Expanded Disability Status Scale; ER, emotion recognition; FSMC, Fatigue Score for Motor and Cognitive Functions; TOM, theory of mind.

MusiQoL global score was 73.9 (*SD* 11), and the subscores were 68.1 (18) for ADL, 73.9 (15) for PWL, 70.1 (15) for SPT, 62.4 (19) for RFr, 73.6 (21) for RFa, 65.5 (26) for SSL, 79.6 (19) for COP, 84 (18) for REJ and 82.2 (16) for RHCS. Accuracy scores of both tasks (ER and TOM) did not correlate significantly with global MusiQoL or its subscores (all *p* > .05).

## 4 DISCUSSION

In our sample, the extent of the social cognition impairment was depending on to the chosen test. Both the emotion recognition and the theory of mind task were influenced by the cognitive function, which was more pronounced in the emotion recognition task. On the other hand, depression, fatigue, and disability were not related to the social cognition ability.

In a meta‐analysis, Cotter and colleagues analyzed the data of the published literature concerning SC in MS until then (Cotter et al., 2016). Comparing ER and TOM tasks, the authors revealed the highest effect size in testing SC impairment for the RMIE task. In our data, the mean accuracy of the RMIE task was 63.6%, which is in between the accuracy range of patients with autism spectrum disorders and healthy controls according to the originally publication (61 vs. 72%, respectively) (Baron‐Cohen et al., 2001). In contrast, accuracy of the ER task was higher, indicating that the RMIE task was more difficult to solve.

There are many theoretical approaches characterizing perceptive and modulatory domains in social cognition (Adolphs, 2006; Beer & Ochsner, 2006; Dalgleish, 2004; Frith & Frith, 2012). The SC tasks investigated here represent perception and modulation unequally. Compared to TOM, ER is regarded as more cognitive demanding (Henry et al., 2015, 2016), and even mild cognitive impairment leads to deficits in emotion recognition (McCade et al., 2011). Our data are in line with these findings, as cognition is the only clinical factor that is related to social cognitive ability in our sample, with a greater extent in ER compared to TOM. It is not surprising that cognitive function is related to both tasks, but here we could show for the first time in one sample that cognition affects ER more than TOM.

Only a few studies focused on the influence of SC impairment on QOL. Phillips and colleagues revealed a relation between deficits in emotion regulation, emotion perception, and the psychological and social dimension of QOL, measured by the World Health Organization Quality of Life Questionnaire (Phillips et al., 2011, 2014). We here could not confirm these results with an internationally validated, multidimensional QOL questionnaire for MS patients (Flachenecker et al., 2011). This was rather unexpected. Comparing the results with the validation studies of the MusiQoL, our patients did have higher QOL scores, even in comparison with the subgroups of the less disabled MS patients (Flachenecker et al., 2011; Simeoni et al., 2008). This only slightly impaired QOL in our sample might be the reason why no correlation could be found in our study. On the other hand, our results in line with a more recent study by Ciampi et al. (2018), who did not find a significant correlation between SC and quality of life in a cohort of patients with progressive MS.

In the study done by Phillips et al., the smaller sample (*n* = 32) of MS patients had cognitive impairments as well (Phillips et al., 2011). Whether the tests used for the assessment of cognitive ability in that study were sufficient (to correct for cognition in their statistic) or whether fatigue, that was not measured, might have influenced the results is rather speculative. All these questions have to be verified in larger samples.

We are aware of some limitations.

First, we did not include a control group. The aim of the study was to investigate influential factors on SC in a sample of MS patients and explore the role of SC decline on QOL. Therefore, a one‐sample multivariate analysis in a MS patient cohort was used.

Second, we here tested a sample of 50 patients with MS with a rather minor severity of the disease with median EDSS of 2.0. For this cohort, we were able to investigate the interacting factors and the impact on SC. The median EDSS is comparable with the meta‐analysis summarizing the SC data so far (Cotter et al., 2016). We are not able to generalize our findings for the entire disease course. As the interaction between the variables disability, depression, fatigue, cognition, and social cognition becomes more complicated with increasing disability, and each factor might affect SC itself in the later disease course, further research should focus on adequate sample sizes.

Finally, we are aware of methodological limitations as well. We here analyzed the disease disability with the EDSS. There is a debate about the validity of the EDSS in detecting the changes in disease progression (Amato & Portaccio, 2007). In a systematic review, Meyer‐Moock and colleagues emphasized a good sensitivity of the EDSS for detecting changes in disability at least for lower scale values, but we are aware of the nonlinearity of the scale in assessing disability (Meyer‐Moock et al., 2014). Cognitive testing was based only on the BRB. Since impaired visual perception could impair the ability of SC in general, a special task for this ability would have been necessary to exclude the systematic error.

Additionally, we only focused on the total BRB score in the assessment of the cognitive demand. A rather detailed association between different aspects of SC, different neuropsychological domains, different disease forms, and other clinical variables like disease duration will be interesting in further investigations on SC in MS.

In conclusion, quantifying SC with ER compared to TOM tasks leads to different results, even in the same cohort of MS patients. Both SC tasks were influenced by cognition, with a higher cognitive demand on identifying rather than assigning emotional states. According to these results, TOM tasks like the RMIE should be used in the assessment of SC in MS because of the higher level of difficulty and the lower influence of cognition on the task. Social cognition ability does not necessarily have an influence on QOL.

## CONFLICT OF INTERESTS

The authors have nothing to declare.

## AUTHOR CONTRIBUTION

All authors made substantial contributions to conception and design of the study. All authors helped in the acquisition, analysis, and interpretation of the data and gave their final approval of the version to be published.

### Peer Review

The peer review history for this article is available at https://publons.com/publon/10.1002/brb3.1925.
